# Residential Segregation and Social Trust of Immigrants and Natives: Evidence From the Netherlands

**DOI:** 10.3389/fsoc.2020.00045

**Published:** 2020-07-07

**Authors:** Conrad Ziller, Christoph Spörlein

**Affiliations:** ^1^Institute of Sociology and Social Psychology, University of Cologne, Cologne, Germany; ^2^Institute of Social Sciences, Heinrich Heine University of Düsseldorf, Düsseldorf, Germany

**Keywords:** ethnic segregation, income segregation, social trust, neighborhood studies, integration paradox

## Abstract

This study examines the relationship between residential segregation and social trust of immigrants and natives in the Netherlands. Building on previous studies that have found evidence for a negative segregation-trust link, we present a nuanced narrative by (i) distinguishing between an ethnic minority and majority perspective, (ii) elaborating theoretical foundations on the moderating role of individual exposure in the form of ethnic minority concentration in the neighborhood, and (iii) taking income segregation into account. In addition to the refined theoretical framework, our study employs a rigorous empirical approach. Using two waves (2009 and 2013) of the Netherlands Longitudinal Lifecourse Study—a geocoded panel study with an oversampling of Moroccan and Turkish immigrants—we are able to study the influence of (changes in) municipality-level segregation patterns for both natives and immigrants, and consider the roles of both neighborhood ethnic minority concentration, as well as income-based segregation. Results from four-level multilevel models show that ethnic segregation is negatively related to the social trust of immigrants. At the same time, this negative relationship is particularly strong in neighborhoods with a low level of minority population concentration, which provides support for the so-called integration paradox where negative intergroup interactions reduce social trust. For respondents of Dutch origin, we find no evidence that their social trust is sensitive to ethnic segregation or that this relationship is conditional on minority concentration at the neighborhood level.

## Introduction

Residential segregation along ethnic lines is a major hurdle to the social integration of immigrants and ethnic minorities. Previous research has shown that, for example, residential segregation is associated with lower levels of generalized social trust (Rothwell, 2012), and that ethnic diversity has a particularly negative impact on social trust in highly segregated residential areas (Uslaner, 2012). A reason for these findings is that living in segregated residential areas prevents residents from experiencing (positive) intergroup contact, which in turn leads to reservations about the other group, perceptions of intergroup threat, and general mistrust (Putnam, 2007; Van der Meer and Tolsma, 2014).

This study examines the role of residential segregation for social trust of immigrants and natives in the Netherlands. While we generally expect that residential segregation is related to reduced trust, we build on previous research in the following three respects. First, the literature on the effects of ethnic diversity or ethnic segregation on trust has largely focused on the general population, or on majority members only (Putnam, 2007; Rothwell, 2012). This has been criticized, as for ethnic minority and majority members, underlying mechanisms might be distinct (Abascal and Baldassarri, 2015). We consequently examine the implications of residential segregation separately for ethnic minority members (here: respondents of foreign origin) and majority members (here: respondents of Dutch origin).

Second, previous research has largely ignored the role of individual exposure, i.e., the “experience of segregation as felt by the average minority or majority member” (Massey and Denton, 1988, p. 287). We conceptualize residential segregation as an uneven distribution of ethnic groups across neighborhoods within a municipality. In a low segregation setting, the minority share in a municipality is evenly distributed across all neighborhoods, whereas the share of minorities is concentrated only in some neighborhoods (but not in others) in a high segregation setting. While the degree of ethnic segregation can be expected to shape overall social experiences in a municipality, we argue that whether, for example, an ethnic minority respondent lives in a neighborhood with a high or a low minority concentration is important. This specification allows for a nuanced theoretical framework of trust development, taking exposure as a relevant moderator variable into account.

Third, according to the ethnic (or racial) proxy hypothesis (Emerson et al., 2001), residential segregation along ethnic lines is regularly conflated with socio-economic disparities. This means that concentrated disadvantage, rather than ethnic segregation could be responsible for the negative consequences of segregation. Given the relevance of economic resources for the development of social trust (Brandt et al., 2015), we disentangle the impact of ethnicity and social status by considering individual and contextual variations in socio-economic resources, as well as an explicit measure of income segregation as competing predictor variables.

All in all, we aim to contribute to the literature by explicitly taking the minority perspective into account, by elaborating on the theoretical mechanisms linking segregation and trust through the inclusion of arguments regarding the interplay of ethnic segregation and ethnic concentration, and by disentangling ethnic segregation from socio-economic segregation.

Empirically, we use two waves (2009 and 2013) of the Netherlands Longitudinal Lifecourse Study, a geocoded panel study with a large immigrant sample, which allows for investigating the role of segregation patterns for both natives and immigrants. Results from multilevel models and two-way fixed effects models show that ethnic segregation is negatively associated with social trust of immigrants. In addition, this relationship is moderated by the extent of minority concentration at the neighborhood-level: individuals of foreign origin living in a low-concentration neighborhood experience a considerably greater reduction in social trust due to segregation compared to those living in a neighborhood with a high minority share. For the Dutch majority, our results do not show a relationship between ethnic segregation and social trust.

## Theoretical Framework

### Ethnic Diversity, Segregation, and Social Trust

Ethnic diversity refers to the composition of a population with respect to the share of one or more minority groups compared to a reference group (e.g., natives, autochthones, or majority group). Apart from basic measures of minority proportions, a widely-used indicator is the fractionalization index that measures the probability that two randomly drawn individuals within a spatial setting are not from the same ethnic group (Tolsma et al., 2009; Uslaner, 2012; Schaeffer, 2013; Ziller, 2015). In contrast, ethnic segregation refers to the spatial distribution of ethnic groups, or “the degree to which two or more groups live separately from one another, in different parts of the urban environment” (Massey and Denton, 1988, p. 282). Hence, ethnic diversity and ethnic segregation might be empirically related, but are conceptually distinct, with diversity dependent on the relative size of the groups being compared, while segregation is not.

A plethora of studies have examined the potential negative consequences of (immigration-related) ethnic *diversity* for social trust, a term which describes the general expectation that (unknown) others will behave in a reliable and just manner, rather than being selfish or acting against one's interests (e.g., Delhey and Newton, 2005; Gundelach and Traunmüller, 2013; Laurence, 2013; Van der Meer and Tolsma, 2014; Ziller, 2015). While the empirical evidence appears to be mixed, a recent meta-analysis (Dinesen et al., 2020) taking more than 80 studies into account, finds a systematically negative relationship between ethnic diversity and social trust. This negative relationship is typically stronger for trust in neighbors and when studying diversity of local areas.^1^ At the same time, several studies find that demographic, economic, political, and cultural characteristics moderate the relationship between ethnic diversity and trust (Kesler and Bloemraad, 2010; Uslaner, 2012; Helbling et al., 2015; Ziller, 2015; Gundelach and Manatschal, 2017; Ziller et al., 2019).

Several arguments have been invoked with regard to the social mechanisms underlying a possible negative link between ethnic diversity and social trust. According to intergroup conflict theory (Blumer, 1958; Esses et al., 1998; Stephan and Renfro, 2002), the presence of outgroup members fosters (perceptions of) intergroup competition for economic resources or cultural dominance, which in turn increases perceived levels of outgroup threat and outgroup distrust. Alternatively, a high concentration of, or an increase in the ethnic minority population might inhibit cooperation across ethnic lines and lead to an increasing impression on the part of residents' that there is a lack of common norms, especially if language barriers exist, or the ethnic minority group is culturally distinct from the majority population. This could result in heightened perceptions of uncertainty and anomie, social withdrawal, and increasing general social distrust, as highlighted in Putnam's (2007) widely recognized “constrict hypothesis,” and related anomie-centered approaches (Tolsma and van der Meer, 2017).

In contrast to conflict approaches, intergroup contact theory (Pettigrew and Tropp, 2011) posits that (immigration-related) ethnic diversity provides an opportunity structure for members of different ethnic groups to interact and connect with each other. Intergroup contact in turn facilitates improving attitudes toward the (ethnic) outgroup and fosters outgroup trust (Stolle and Harell, 2013; Gundelach and Freitag, 2014). Social interactions with outgroup members might also enable the development of generalized social trust (Blau and Schwartz, 1984; Glanville et al., 2013). The reason for this is that through social interactions with people from different backgrounds, individuals can learn about the motives of others and consequently begin to perceive the social world as more predictable, and thus less threatening (Hardin, 2002), and fostering the development of complex and inclusive social identities (Wenzel et al., 2003; Schulz and Leszczensky, 2016).

It has been argued that ethnic residential *segregation*, as a measure of unevenness in the spatial distribution of ethnic group members across living areas, maps underlying theoretical mechanisms more effectively than measures of ethnic diversity or minority concentration (Rothwell, 2012; Laurence, 2017). According to intergroup contact theory, residential segregation along ethnic lines impedes opportunities for interethnic contact, resulting in lower levels of trust directed at outgroups, and social trust in general.

As an extension, segregation has also been conceived of as a moderator that triggers the extent to which threat effects outweigh contact effects, and *vice versa* (Laurence et al., 2019). In a similar vein, Uslaner (2012, p. 15) concluded from empirical analyses carried out in multiple Western countries that “segregation rather than diversity drives down trust,” and that “the positive effects of living in an integrated community with friends of diverse backgrounds outweigh any negative impacts of heterogeneity.”

While it is plausible to assume residential segregation to be consequential for (intergroup) social contact, it might affect minority and majority members differently regarding the way they feel socially integrated, and perceive fellow citizens as being principally trustworthy.

### Taking the Ethnic Minority Perspective Into Account

**Figure 1:**
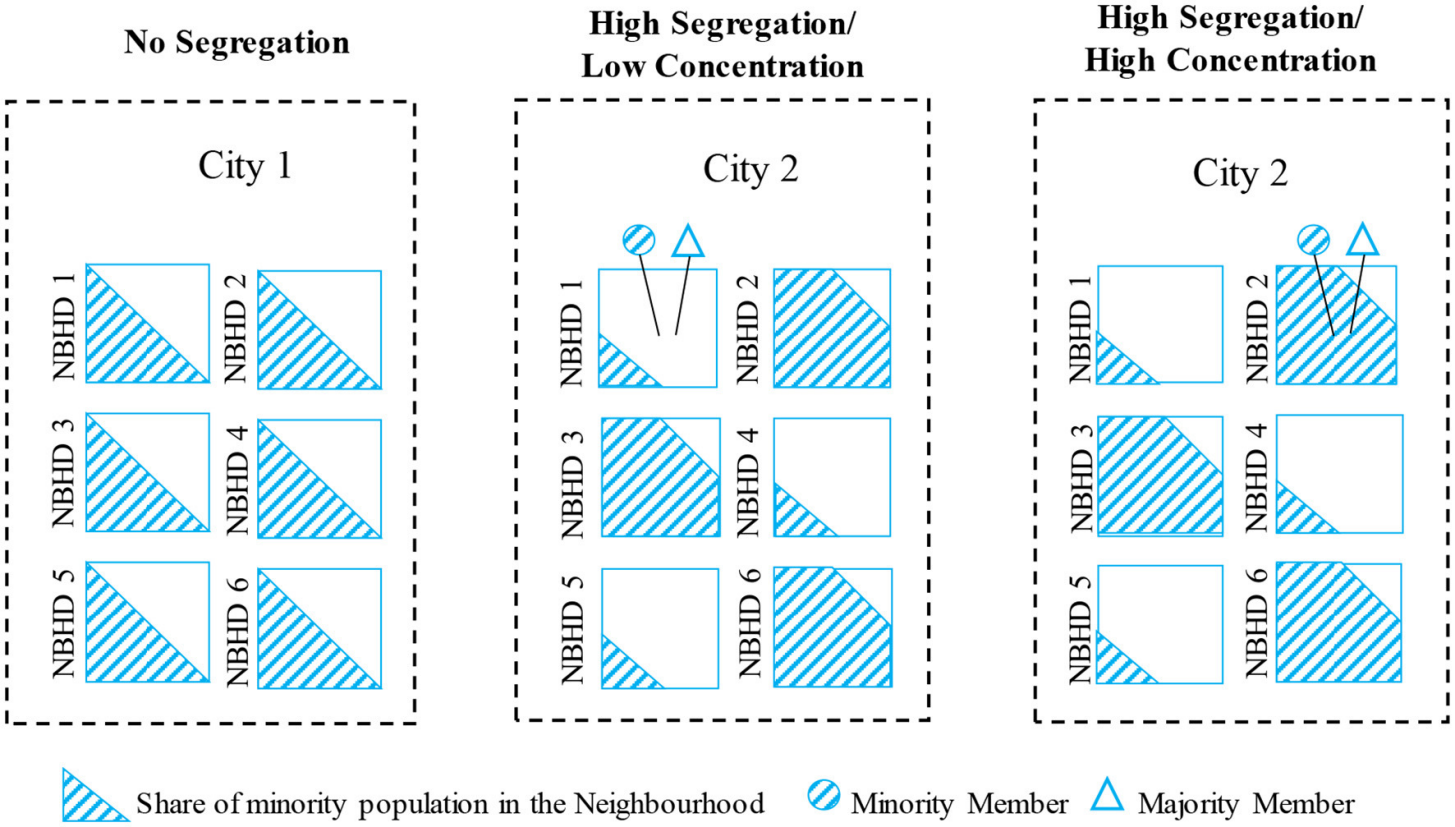
Ethnic segregation and concentration. NBHD, Neighborhood.

Most empirical studies on the relationship between ethnic diversity or ethnic segregation and social trust have examined this empirical relationship in the overall or majority population (Rothwell, 2012; Laurence, 2017). In general, ethnic diversity or minority concentration has a clear distinct implication to majority members (i.e., more outgroup neighbors) and minority members (i.e., more ingroup neighbors). Adding the perspective of ethnic segregation, it is useful to simultaneously take the analytical levels of municipalities and neighborhoods into account. At the municipality level, higher rates of segregation mean higher propensities of ingroup contact, on average. However, group-specific effects may vary depending on the actual minority concentration in immediate living areas. Figure 1 illustrates relevant combinations of municipality segregation by neighborhood concentration.

The left-hand panel in Figure 1 shows a city in which the minority population residing in a municipality (City 1) is evenly distributed across neighborhoods, which indicates a minimal degree of ethnic segregation. Note that the within-neighborhood shadings illustrate only the share of the minority proportion resident in an area, and do not convey information about the spatial distribution within a neighborhood. The middle and right-hand panels show a highly segregated city (City 2) and randomly selected individuals from the minority and majority group. In the middle panel, the two sampled individuals live in a neighborhood setting with a low minority concentration, while in the right-hand panel, the individuals reside in a high-concentration neighborhood. Hence, although both situations relate to one city with one level of residential segregation, the immediate neighborhood environment is starkly different for the individuals from the middle panel compared to those sampled in the right panel. The middle panel effectively represents a situation where nominal levels of segregation are high, but the sampled immigrant lives in a low-concentration environment, sharing his or her immediate surroundings mostly with majority members. The opposite is true for the right panel, where the sampled majority individual resides in an area where they are effectively in the minority^2^.

In terms of theory, we have strong reasons to suspect differential effects on social trust across the respective constellations. With reference to minority members, (i) assimilation theory suggests that living in segregated areas prevents them from having social interactions with majority members (or members of the receiving society in case of immigrants), which in turn hampers their social integration. Ethnic segregation might also stimulate ethnic discrimination (Winter and Zhang, 2018; Zhang et al., 2019). Hence, we expect residential segregation to inhibit social dimensions of minority integration which in turn hampers the development of minorities' social trust (Hypothesis 1). Looking at patterns of exposure, the negative impact of residential segregation on social trust is expected to be particularly strong for minority members living in neighborhoods characterized by a high minority concentration (H1a). In contrast, minority members living in areas with a low minority concentration have higher incentives to assimilate, which should mitigate the negative context effect of municipality segregation (H1b).

A contrasting theoretical perspective to assimilation is (ii) the immigration paradox. Using data from several Western countries, Uslaner (2012) finds that while both natives and immigrants have higher trust levels when living in integrated (i.e., less segregated) residential areas, ethnic minorities or immigrants hardly benefit from intergroup contact with natives in terms of generalized social trust, even if they live in less segregated areas. This implies diverging mechanisms for majority and minority populations when it comes to converting social contact into social trust—possibly because increased contact with members of the receiving society exposes immigrants to additional occasions of discrimination and unequal treatment, as highlighted in works on the integration paradox (Verkuyten, 2016). From this perspective, it can be expected that the relationship between ethnic segregation and minority trust is positive, meaning that high levels of residential segregation lead to high levels of social trust, and vice versa (Hypothesis 2). To elaborate on this theoretical assumption, particularly minority members who live in high-concentration neighborhoods are expected to develop high levels of social trust because they may circumvent negative experiences of rejection or discrimination that are likely to occur as a result of direct contact with majority members (H2a)^3^. In contexts of low concentration, potentially negative experiences are much more likely to occur which should lead to a negative moderation of a positive segregation effect (H2b).

For majority members and those living in municipalities that are largely separated from ethnic minorities, we can employ arguments informed by (iii) intergroup contact theory (Pettigrew and Tropp, 2011). According to earlier research on diversity and segregation effects, a high degree of residential separation may impede intergroup contact, heighten the salience of group boundaries, and increase the potential of intergroup conflict (Legewie and Schaeffer, 2016). In turn, this should lead to an overall reduction in generalized social trust (Hypothesis 3). At the same time, this effect should be contingent upon the particular living environment. People in areas of high concentration are expected to nonetheless profit from intergroup contact experiences in terms of social trust (positive interaction, H3a), while for those in low-concentration settings, the negative segregation effect is reinforced instead (H3b).

In contrast to the standard intergroup contact and conflict narrative, it is also plausible to assume conflict-mitigating effects for majority members as highlighted in recent research on the possible beneficial effects of segregation (Light and Thomas, 2019). From this perspective, living in a segregated community improves majority members' social trust as it attenuates conflictual experiences of intergroup contact (Hypothesis 4). It should be emphasized here that this is a plausible narrative on the condition that ethnic inequality is present, and intergroup contact is largely negative, which offsets intergroup contact's prejudice-reducing and trust-enhancing potential (Barlow et al., 2012). Taking experiential settings of neighborhoods into account, we assume that a positive segregation effect is mitigated or reversed (negative moderation) for individuals who live in areas with a highly concentrated minority population (H4a), while avoidance of negative intergroup contact is a feasible option, on average, for those living in a setting with a low concentration of minority population (H4b).

**Figure 2:**
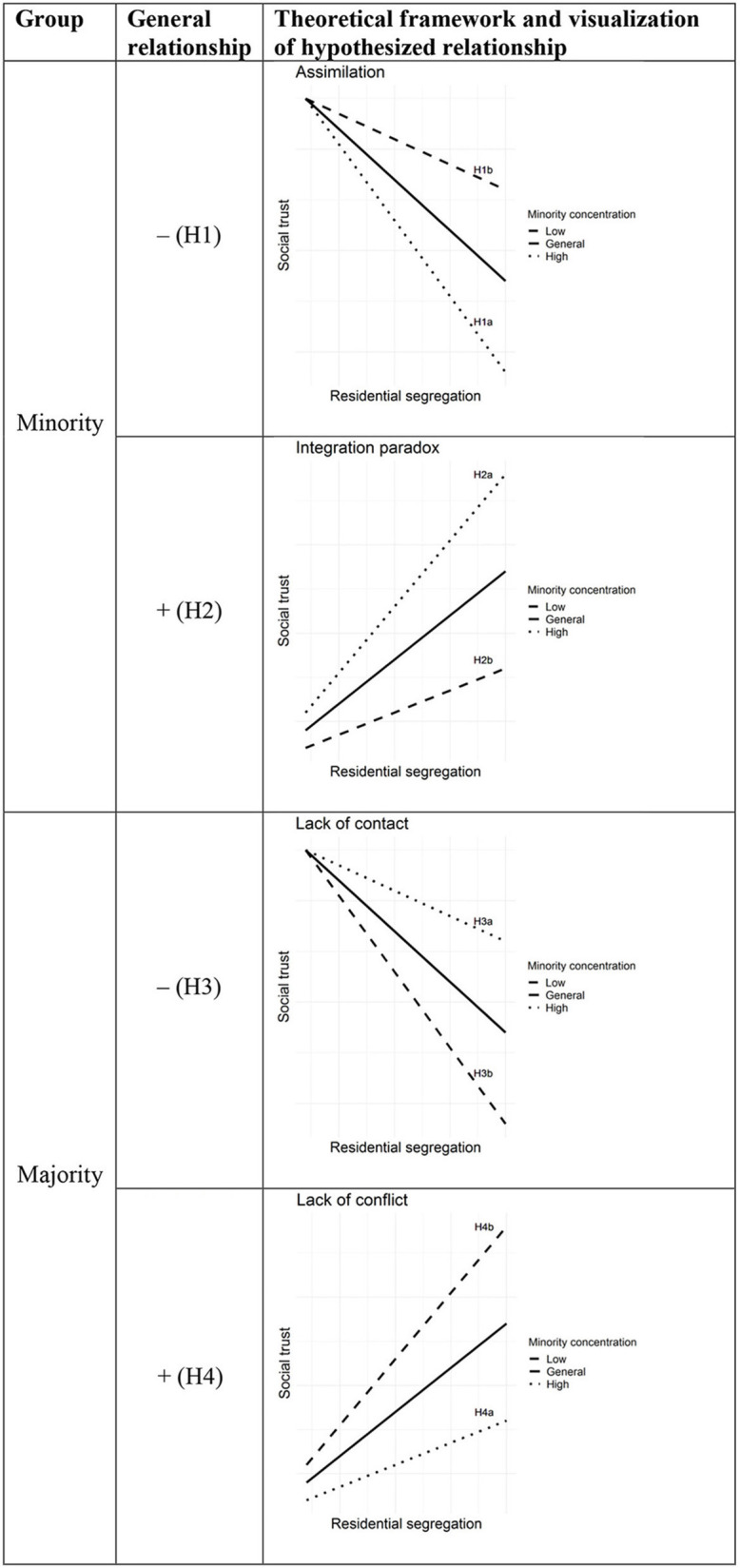
Graphical illustration of hypotheses.

Figure 2 provides a concise overview of the expected relationships based on the competing theoretical ideas, as well as separately for majority and minority groups.

### Disentangling Ethnicity and Status: The Role of Income-Based Residential Segregation

In addition to social contact dimensions, ethnic segregation also reflects socio-economic inequalities, where residential areas with a high concentration of immigrants or ethnic minority members also constitute areas of high socio-economic disadvantage (Teltemann et al., 2015). In other words, ethnic residential segregation may not solely be driven by preferences for ingroup contact, or discriminatory renting practices by native-majority landlords, but rather as a function of immigrants' resources. Due to their on average lower positions on the labor market, and the resulting lower labor market returns in the form of income and status (Luthra, 2013), immigrants essentially have a systematically different set of potential accommodation options, thus leading to their clustering in certain areas of cities where average rents are lower. Conversely, natives, whose average income levels are often considerably higher, are less constrained in this respect and are thus able to avoid the low-rent districts. For example, Spörlein and Schlueter (2018) demonstrate that roughly 25 percent of ethnic segregation patterns within a German city can be explained by the systematic differences in socio-economic resources that immigrants and natives have at their disposal. A further 25 percent are accounted for the local pricing structure as a contextual indicator of the opportunity structure for low resource individuals. Like others before us, we therefore invoke the argument that economic and preference considerations are closely tied together, and need to be disentangled to reach systematic conclusions about the role of segregation (Leckie et al., 2012; Spörlein and Schlueter, 2018).

Stressing the relevance of arguments subsumed under the ethnic (or racial) proxy hypothesis does not negate the fact that preferences and discriminatory practices are irrelevant in generating segregation patterns (Clark, 1986). While economic factors are a major explanation for ethnic segregation, there is also ample evidence that majority members greatly prefer lower rates of outgroup presence compared to minority members. Similarly, research on the so-called “White flight” suggests that social preferences, as well as concerns related to crime and security, account for majority members moving out of diversifying neighborhoods, which additionally fuels residential segregation along ethnic lines (Emerson et al., 2001). Nonetheless, disentangling ethnic from economic or resource-based segregation seems paramount because these two dimensions strongly overlap, and resource-based segregation may confound the relationship between ethnic segregation and trust.

## Data and Methods

### Data and Variables

To test our hypotheses, we use the first and second wave of the Netherlands Longitudinal Life Course Study (NELLS; Tolsma et al., 2014). The NELLS data survey data includes measures relevant to the proposed theoretical relationships, contains an oversample of the two large ethnic minority groups in the Netherlands (Turks and Moroccans), and allows for constructing segregation indices thanks to it featuring geo-codes at the level of neighborhoods and municipalities. Data collection was based on a random sample of 35 municipalities stratified by region and level of urbanization (including the four biggest cities Amsterdam, Rotterdam, Den Haag, and Utrecht). Subsequently, respondents (between 15 and 45 years of age) were randomly selected from population registries. Moroccan and Turkish individuals were oversampled. However, the sample was restricted to more urban areas, due to the low number of Moroccans and Turkish people living in rural areas. Hence, the population frame represented by the NELLS data refers a population in the Netherlands that tends to be young and urban. The interview was carried out face-to-face in the first wave, and in the second wave, interviews took place either in a face-to-face setting or via a web survey.

We include only respondents in the analytical sample that were interviewed at both time points (the first wave was fielded between December 2008 and May 2010, and the second wave between June and August 2013), and had not changed their place of residence between waves^4^. Since we are interested in group specific effects, we distinguish between respondents of Dutch origin (“natives”) and those of foreign origin (“immigrants”). A person is classified as being of Dutch origin if both parents were born in the Netherlands, while a person is classified as being of foreign origin if the person and one, or both parents, were born outside the Netherlands, as well as if the person was born in the Netherlands and one, or both parents, were born in another country. We also present results separately for foreign-born immigrants (“first generation”) and people of immigrant descent (foreign-born mother and/or father, so-called “second generation”) in the online appendix (Tables A8, A9).

As outcome variable, the NELLS survey offers several items on social trust. These are (i) “Nowadays you really do not know who you can trust,” (ii) “Most people are disappointing when you get to know them better,” (iii) “Most people can be trusted,” (iv) “You can't be too careful enough with other people,” (v) “If you are too trusting, people will use you,” and (vi) “If you help others, you will often be cheated on” (all measured on a 5-point Likert-type scale). Besides their theoretical relevance, selected items need to fulfill the following empirical criteria in order to reflect meaningful indicators and to build a valid index to be used in empirical analysis: (1) Items are required to vary at the individual level both between individuals and over time, (2) items are required to display variation at the municipality (and municipality-time) level in order to be explained by differences in municipality (over-time changes in) segregation, (3) items need to be empirically connected to form a coherent index, and (4) they need to be invariant across ethnic groups and time in order to be comparable.

Firstly, we computed—for respondents interviewed in both waves—intra-class correlations where within-individual observations over time are nested within individuals, municipalities, and municipality-waves. This provides information about the proportion of variance that can be attributed to the level of individuals, municipalities, or municipality change between waves^5^. While all items display substantial over-time change within individuals (i.e., 1–ICC_individuals_), only items (i), (ii), and (vi) show variance at the municipality and municipality-year level close to five percent. Next, we look at the inter-item correlation and find Pearson's r correlations of between 0.34 and 0.62 (all statistically significant). An index of the recoded (i.e., higher values indicate higher trust) items (i) “Nowadays you really do not know who you can trust,” (ii) “Most people are disappointing when you get to know them better,” and (vi) “If you help others, you will often be cheated on” shows sufficient consistency (Cronbach's α = 0.78).

Finally, we test the extent to which the three items are comparable across ethnic groups and survey waves using multigroup confirmatory factor analyses (Davidov et al., 2014; and see the Online Appendix for detailed results). The results demonstrate scalar invariance over time, and partial scalar invariance between natives and immigrants, which means that latent variable scores can be compared across groups and can be used in regression analyses. Thus, the outcome variable in the empirical models below consists of latent factor scores obtained from a confirmatory factor analysis of the three trust items ([i], [ii], and [vi]), which were then linearly rescaled to range between 1 (“low trust”) and 5 (“high trust”) to facilitate interpretation^6^.

The core predictor variable is residential segregation. We use the index of dissimilarity, one of the most prominent measures of residential segregation (Duncan and Duncan, 1955; Massey and Denton, 1988), which measures the extent of unevenness in the distributions of two groups over units (i.e., geographical units such as neighborhoods or districts, as well as other units such as occupations or fields of study). To do so, we use municipality-level and neighborhood-level data on proportions of individuals of Moroccan, Turkish, Antillean, Surinamese, other non-Western, and Western origin (i.e., “minority group”), as well as proportions of people of Dutch origin (i.e., “majority group”)^7^. Formally, the index is defined as

(1) $$\begin{array}{r} D=\;\frac{1}{2}\;\left(\sum_{j=1}^{J}|\frac{a_{j}}{A}-\frac{b_{j}}{B}|\right), \end{array}$$

where a_j_ (b_j_) refers to the number of individuals from the minority (majority) group in unit j, and A (B) to the total number of individuals in the minority (majority) group. Values of D are bounded by 0 (completely integrated) and 1 (fully segregated), and can be interpreted as the proportion of individuals who would have to change units in order to achieve an even distribution across those units^8^. More specifically in our case, a value of 0.21 (in wave 2) would indicate that 21% of foreign-born or Dutch individuals would have to move to a different neighborhood to achieve an even distribution of foreign-born and Dutch individuals across all neighborhoods. We calculate the dissimilarity index for each of the municipalities in the sample separately based on the neighborhoods that constitute them.^9^ As an additional control variable, we also calculate the extent of income segregation across municipalities. To do so, we categorized the available income information into low (below or equal to a personal income of 19,200 Euros per year, which corresponds to the lowest 40 percent of the income distribution), and high (above 19,200 Euros) income levels^10^.

While ethnic and income segregation are strongly correlated across municipalities (Wave 1: Pearson's *r* = 0.61, *p* = 0.001, *n* = 26; Wave 2: Pearson's *r* = 0.39, *p* = 0.047, *n* = 26)^11^, difference scores between waves are virtually uncorrelated (Pearson's *r* = −0.08, *p* = 0.683, *n* = 26) implying that cross-sectional and longitudinal results might vary due to different underlying mechanisms, or confounding. We therefore provide both cross-sectional and longitudinal fixed-effects regressions.

To empirically assess the role of intermediary variables that connect residential segregation and social trust, we include separate measures of intergroup contact (when looking at natives), and intergroup contact and ethnic discrimination (when looking at immigrants). Intergroup contact is measured as an index for an individual's contact in their neighborhood, at work, and at leisure clubs with people of Turkish, Moroccan, and Surinamese background (for respondents of Dutch origin), and for an individual's contact in their neighborhood, at work, and at leisure clubs with Dutch people (for respondents of foreign origin)^12^. Discrimination is measured as negative experiences across a variety of occasions (i.e., application for job or internship; in the workplace; at school, in class; in the streets, in shops, on public transport; organizations, clubs, sports; and nightlife, nightclubs). We computed a dummy variable which takes a value of 1 if respondents report to have experienced discrimination in at least one of these areas (at least once in a while), and 0 if they report no experiences of discrimination.

We control for several individual and municipality level variables that potentially confound the relationship between segregation and trust. Specifically, we use age in years, occupational status (dummy variable, 1 = unemployment), income (household income before taxes, 16-point scale of income categories), and home ownership (dummy variable, 1 = owner). As macro-level control variables, we include proportions of foreign-born immigrants at the neighborhood level, and average income per capita at the municipality level. We present descriptive statistics on variables employed in the online appendix.

To distinguish neighborhoods with low versus high levels of ethnic minority concentration, we group-centered the neighborhood immigrant share variable at the corresponding municipality means. This allows high and low concentration neighborhoods to be identified in each municipality. In order to test hypotheses on the moderating relationship between ethnic segregation and concentration, we split the samples into high and low concentration neighborhoods, thereby avoiding the use of three-way interaction effects that are difficult to interpret. Note that centering at the overall mean (i.e., looking at high and low concentration neighborhoods that are not necessarily within the same municipality) leads to similar results, as reported below.

To summarize, ethnic segregation is measured at the municipality level using the dissimilarity index calculated across constituting neighborhoods, whereas ethnic concentration represents the share of immigrants at the neighborhood level.

### Methods

In order to test our hypotheses, we first employ a four-level multilevel model where observations over time are nested within individuals, which are again situated in neighborhoods, and municipalities. Second, we use two-way (person and time) fixed effects models that include cluster-robust standard errors at the level of municipality-years to additionally account for clustering and heteroscedasticity. Fixed effects models produce more credible coefficient estimates as they control for all time-constant variations that may be unobserved, and confound the relationship under study (Allison, 2009). The person fixed effects simultaneously absorb all time-constant variations across municipalities, and the time fixed effects account for temporal trending in the outcome variable. Fixed effects models are a quite constrictive approach prone to wiping out the variation necessary to separate signal from noise. Hence, we employ both a flexible multilevel, and a fixed effects approach, and discuss similarities and differences.

It is noteworthy that (fixed effects) regression models assume a correct modeling of the causal order, and that reversed causality would bias estimates (Vaisey and Miles, 2017). We argue that a causal effect from segregation to trust is more realistic (than the other way around) given the pertinence of structural conditions responsible for determining patterns of residential segregation (Lesger and Van Leeuwen, 2011; Grigoryeva and Ruef, 2015). These include features of the physical environment (e.g., location within a city, buildings history, access to transportation), the distribution of employment opportunities, and historical patterns of ethnic diversity or immigrant concentration. Moreover, quasi-experimental evidence from the demolition of public housing demonstrates that changes in residential settings related to the presence of ethnic outgroups have a causal effect on political behavior and underlying social and political attitudes (Enos, 2016). Together, these arguments strengthen our confidence in the reasoning that segregation precedes social trust.

To map our theoretical framework, we present models separately for respondents of Dutch origin, as well as respondents of foreign origin. In addition, we present tests for high and low minority concentration settings separately.

## Results

Table 1 presents results from a cross-sectional multilevel model for minority respondents. The first column presents evidence for our main hypotheses. Accordingly, Model 1 shows a negative effect of ethnic segregation at the municipality level on social trust, supporting the reasoning of assimilationist arguments, which highlight the importance of interethnic contact in fostering social trust (H1). In terms of effect size, moving from the least to the most segregated municipalities is associated with a decrease of −0.38 in social trust (roughly half a standard deviation of the trust variable). Including indicators of intergroup contact and discrimination experienced in Model 2 leads to a reduction in the coefficient magnitude of ethnic segregation (now statistically not significant). This provides evidence that the included factors (especially intergroup contact) mediate the relationship between ethnic segregation and social trust. Including income and income segregation (Model 3) does not lead to a reduction but instead leads to an increase in the coefficient of segregation, which points to a suppression effect (i.e., segregation becomes more systematic for predicting social trust once differences in municipality economic status are accounted for). Apart from these core variables, we find negative (and statistically significant) associations for neighborhood proportions of immigrants, age, and unemployment. In contrast, income and house ownership is positively (and significantly) related to social trust.

**Table 1:** Four-level multilevel regression results respondents of foreign origin.

	**(1)**	**(2)**	**(3)**	**(4)**	**(5)**
	**All neighborhoods**	**All neighborhoods (with mediators)**	**All neighborhoods (with mediators & economic status)**	**Low concentration neighborhoods**	**High concentration neighborhoods**
Ethnic segregation (munic.)	−0.737^*^	−0.569	−0.732	−1.884^*^	−0.425
	(0.375)	(0.326)	(0.380)	(0.911)	(0.408)
Prop. immigrants (neigh.)	−0.008^**^	−0.008^**^	−0.009^**^	−0.015	−0.006
	(0.002)	(0.002)	(0.002)	(0.011)	(0.003)
Age	−0.010^**^	−0.009^*^	−0.009^**^	−0.003	−0.012^**^
	(0.004)	(0.003)	(0.004)	(0.006)	(0.004)
Contact with Dutch		0.047^**^	0.048^**^		
		(0.018)	(0.018)		
Discrimination		−0.072	−0.074		
		(0.045)	(0.045)		
Income	0.048^**^	0.048^**^	0.047^**^	0.039^*^	0.048^**^
	(0.011)	(0.010)	(0.010)	(0.016)	(0.014)
Unemployed	−0.137^*^	−0.112	−0.123^*^	−0.132	−0.167^*^
	(0.059)	(0.058)	(0.059)	(0.095)	(0.075)
House ownership	0.128^*^	0.122^*^	0.127^*^	0.173	0.099
	(0.059)	(0.059)	(0.059)	(0.096)	(0.075)
Av. income (munic.)			0.043		
			(0.036)		
Income segregation (munic.)			0.935		
			(0.704)		
Wave 2	0.045		−0.080	0.121	0.011
	(0.043)		(0.089)	(0.071)	(0.056)
Constant	3.194^**^	2.990^**^	2.174^**^	3.186^**^	3.163^**^
	(0.137)	(0.175)	(0.721)	(0.270)	(0.191)
Random effect municipality	0.188^**^	0.182^**^	0.183^**^	0.227^**^	0.145^**^
Random effect neighborhood	0.047	0.061	0.045	0.284^**^	0.000^**^
Random effect individual	0.494^**^	0.492^**^	0.491^**^	0.469^**^	0.461^**^
Residual	0.609^**^	0.607^**^	0.606^**^	0.556^**^	0.636^**^
N_Municipalities_	26	26	26	21	17
N_Neighborhoods_	140	140	140	68	72
N_Respondents_	617	617	617	224	393
N_observations_	1,234	1,234	1,234	448	786

*Standard errors in parentheses*.

* *p < 0.05*,

** *p < 0.01 (two-sided test)*.

Models 4 and 5 re-estimate Model 1 for a subset of respondents in order to explore the differential association between ethnic segregation and social trust conditional on minority concentration at the neighborhood level. Contrary to hypotheses 1a and 1b, results show that residing in a neighborhood with low concentrations of immigrant residents is associated with considerably lower levels of social trust. For minority respondents living in neighborhoods of high minority concentration (Model 5), the results show a negative, yet compared to Model 4, smaller association that is, however, not statistically significant. Thus, instead of supporting assimilation arguments, these results are in line with H2a and H2b that correspond to the immigration paradox—according to which exposure to the native population is accompanied with increasing potential for conflict.

We now turn to the results for native-born respondents reported in Table 2. The findings reported here do not provide evidence for H3 and H4. Instead, the coefficient estimate is indistinguishable from 0 (b = −0.072, *p* = 0.804). Consequently, an inclusion of potential mediator variables in Models 7 and 8 do not lead to a substantial change in the estimated relationship. In terms of control variables, we find that age and unemployment are negatively and statistically significantly related to social trust, while income and house ownership yield positive and significant associations. Looking at the relationship conditional upon neighborhood minority concentration (Models 9 and 10), we find that in neither low nor in high concentration environments is a systematic relationship between ethnic segregation and social trust apparent in the data.

**Table 2:** Four–level multilevel regression results dutch respondents.

	**(6)**	**(7)**	**(8)**	**(9)**	**(10)**
	**All neighborhoods**	**All neighborhoods (with mediators)**	**All neighborhoods (with mediators & economic status)**	**Low concentration neighborhoods**	**High concentration neighborhoods**
Ethnic segregation (munic.)	−0.072	−0.064	−0.061	−0.209	0.089
	(0.291)	(0.291)	(0.286)	(0.344)	(0.552)
Prop. immigrants (neigh.)	0.001	0.001	0.000	0.001	0.001
	(0.002)	(0.002)	(0.002)	(0.006)	(0.005)
Age	−0.006^*^	−0.006^*^	−0.006^*^	−0.006	−0.004
	(0.003)	(0.003)	(0.003)	(0.003)	(0.006)
Contact with non–native		−0.010	−0.010		
		(0.013)	(0.013)		
Income	0.035^**^	0.035^**^	0.034^**^	0.025^**^	0.052^**^
	(0.007)	(0.007)	(0.007)	(0.009)	(0.015)
Unemployed	−0.137^*^	−0.136^*^	−0.137^*^	−0.107	−0.166
	(0.068)	(0.068)	(0.068)	(0.073)	(0.170)
House ownership	0.202^**^	0.200^**^	0.198^**^	0.161^*^	0.211^*^
	(0.056)	(0.056)	(0.056)	(0.067)	(0.105)
Av. income (munic.)			0.032		
			(0.021)		
Income segregation (munic.)			0.757		
			(0.469)		
Wave 2	−0.005	−0.000	−0.081	0.008	−0.035
	(0.031)	(0.032)	(0.051)	(0.037)	(0.064)
Constant	3.308^**^	3.325^**^	2.716^**^	3.397^**^	3.124^**^
	(0.108)	(0.110)	(0.406)	(0.134)	(0.278)
Random effect municipality	0.127^**^	0.127^**^	0.101^**^	0.120^**^	0.095^**^
Random effect neighborhood	0.000^**^	0.000^**^	0.000^**^	0.074^*^	0.000^**^
Random effect individual	0.518^**^	0.518^**^	0.518^**^	0.530^**^	0.482^**^
Residual	0.472^**^	0.471^**^	0.472^**^	0.445^**^	0.537^**^
N_Municipalities_	26	26	26	21	16
N_Neighborhoods_	160	160	160	105	55
N_Respondents_	795	795	795	595	200
N_observations_	1,590	1,590	1,590	1,190	400

*Standard errors in parentheses*.

* *p < 0.05*,

** *p < 0.01 (two-sided test)*.

### Supplementary Analyses

In addition to the cross-sectional multilevel models, we present findings from longitudinal fixed effects models in Tables A6, A7 in the online appendix. The results show how changes in contextual ethnic segregation relate to changes in social trust. With reference to respondents of foreign origin, the general relationship between ethnic segregation and social trust (Model A1) is negative and highly significant, and again supports the assimilation view of segregation in that an increase in the level of ethnic segregation reduces social trust of minority individuals (H1). Here, neither changes in contact with Dutch individuals or discrimination (Model A2), nor average income or income segregation (Model A3) can systematically account for changes in social trust levels. In terms of maximum effect size, we see a social trust level −0.51 lower in the most than in the least segregated areas of residence. Apart from segregation, individual income and age are systematically related to changes in social trust. Taken together, the fact that there is no systematic relationship between changes in interethnic contact and discrimination, as well as in income-related factors and changes in social trust, suggests that the theoretical mechanisms linking ethnic segregation to social trust can plausibly explain differences across, rather than within, individuals.

Models A4 and A5 replicate the results pattern found in the multilevel analyses. Here, the coefficient estimate in the low concentration setting is considerably higher than that found in the high concentration setting, although only the latter is statistically significant, which likely is due to the higher number of observations (and statistical power) in this group. Hence, also the longitudinal results provide evidence in support of H2a and H2b, and the integration paradox.

Looking at respondents of Dutch origin (Table A7), and just as in the multilevel models, we find no systematic relationship between ethnic segregation and social trust in general, as well as no systematic results pattern when comparing low and high concentration neighborhoods.

In a next step, we estimate empirical models for first and second generation immigrants. The results are presented in Tables A8, A9 in the online appendix. While we find no systematic association for the first generation, the relationship is remarkably strong when looking at second-generation respondents (Table A9). Here, we find a strong negative association between segregation and trust, which is mediated particularly by discrimination. Moreover, we find strong evidence that the relationship is strongest in neighborhoods of low minority concentration (i.e., support for H2a and H2b).

Finally, we estimate models based on Moroccan and Turkish respondents (the two largest immigration groups in the Netherlands) and measures of neighborhood minority concentration based on proportions of the respective ingroup residing in a given geographical area (Moroccan or Turkish). The results are presented in Tables A10, A11 in the online appendix and show similar patterns to those reported in the main models for Moroccan, but not Turkish respondents. The fact the social trust of Turkish respondents is unsystematically related to segregation suggests that this group exhibits similar patterns as in the native population. Nonetheless, additional research is needed to theoretically specify and test immigrant group differences when it comes to segregation effects.

## Conclusion

In this article, we rely on high-quality data at the individual and contextual level to investigate, first, whether individuals living in highly segregated municipalities differ in their social trust from those living in less segregated context and, second, to what extent minority concentration (as a measure of exposure) moderates how ethnic residential segregation relates to social trust. We stated theoretical expectations separately for minority members and natives. Overall, our findings show a robust negative association between ethnic segregation and social trust for people of foreign origin. This overall pattern supports an assimilationist perspective on segregation and trust. However, looking at specific neighborhood conditions, we also find support for the so-called integration paradox: people of foreign origin are observed to hunker down (in terms of social trust) if they live in a generally segregated municipality and—at the same time—in a neighborhood with few co-residents of foreign origin.

Additional findings from models based on first and second generation immigrants, as well as mediator variables, complement the picture. The negative association between segregation and trust (which is re-enforced in low concentration neighborhoods) is driven by second generation immigrants. Moreover, experienced discrimination appears to critically mediate this relationship for this group. Essentially, this means that segregation decreases the social trust of people with foreign-born parents who are themselves born in the Netherlands because they experience discrimination. This particularly occurs for those who live in contexts predominantly populated by Dutch natives, which quite accurately represents the integration paradox. According to this narrative, respondents with an immigration background feel less integrated because they are more exposed to natives and thus experience more discrimination and opportunity gaps compared to natives (Verkuyten, 2016; Schaeffer, 2019; Ziller and Heizmann, 2019).

That fact, that results are driven by second generation immigrants also points to another important aspect regarding the mechanisms underlying the purported relationships. What this result may show is that for mechanisms related to perceived discrimination to take hold, immigrants need to possess the cultural resources (e.g., linguistic skills) to decode discriminatory aspects of intergroup interactions in the first place—resources which are on average more prevalent among second generation compared to first generation individuals.

Results from longitudinal fixed effects models also corroborate how minority individuals living in more segregated municipalities express less social trust, by relating over-time changes in segregation with over-time changes in social trust. While the neighborhood context appears to moderate how ethnic segregation translates into social trust of immigrants, we at the same time, find from the multilevel models that the variation at the neighborhood level is quite small (compared to the individual or municipality level) once the municipality context is taken into account. This is a potential reason for why previous research which has focused on neighborhood effects using NELLS data has found only limited evidence for contextual effects (De Vroome et al., 2013).

In contrast to previous studies on segregation effects, which mainly focused on responses from the general population (Rothwell, 2012; Uslaner, 2012; Laurence, 2017), we find no indication for trust-erosion among the native Dutch population. This is in line with previous research, which finds no systematic association between ethnic diversity and social trust in the Netherlands (Tolsma et al., 2009; De Vroome et al., 2013). Apart from specific features of the Netherlands (e.g., population structure, institutional or historical factors), a possible reason is that the sample we use represents a rather young and urban population, the kind that is typically more cosmopolitan and pro-immigration compared to older and more rural segments of the population (Maxwell, 2019). However, even though we did not find any systematic evidence that ethnic segregation hampers the social trust of natives, this does not imply there are no effects on other dimensions relevant for intergroup relations, such as social distance or forms of social conflict.

Despite the careful empirical strategy we exercised, we nonetheless would like to suggest two avenues for future research on the social consequences of residential segregation. First, studying the group of respondents who moved neighborhoods or municipalities between survey waves would enable researchers to assess whether this group is indeed more sensitive to changes in contextual conditions, and whether the decision to move is a result of, or an indication of eroding social trust. This would also provide strong evidence in support of segregation being a causal factor in the decline of social trust (rather than vice versa), which could not be empirically determined with certainty here, given that the panel data we use in this study comprises two waves only. Second, the advent of available register-based data opens the possibilities of measuring segregation in fine-grained and continuous ways which will additionally improve the examination of individual exposure to ethnic segregation.

## Data Availability Statement

Publicly available datasets were analyzed in this study. This data can be found here: https://easy.dans.knaw.nl/ui/datasets/id/easy-dataset:59831.

## Ethics Statement

Ethical review and approval was not required for the study on human participants in accordance with the local legislation and institutional requirements.

## Author Contributions

CZ and CS designed research and wrote the paper. CZ performed research and analyzed data. All authors contributed to the article and approved the submitted version.

## Conflict of Interest

The authors declare that the research was conducted in the absence of any commercial or financial relationships that could be construed as a potential conflict of interest.

## Footnotes

^1^Apart from this trend, heterogeneous research findings also exist with regard to the role of neighborhood conditions in general. For example, De Vroome et al. (2013) find only limited evidence for an empirical relationship between neighborhood conditions and social trust.

^2^It is important to note that conceptually within-neighborhood residential patterns (e.g., at the level of streets or blocks) might also be relevant. However, with the data available we can only approximate individual experiences of the factors evenness and exposure at the level of municipalities and neighborhoods, respectively. We consider the immediate living environment of individuals as a relevant experiential setting, but also want to emphasize that other settings in which people socially interact are also relevant, including the workplace, associations and sport clubs, supermarkets, and public transport. These settings are typically not within the same housing block or street where people live, but are likely to be within the same neighborhood or municipality.

^3^Nonetheless, possible positive consequences of living in an ethnic enclave are likely to occur with respect to trust in particular others (i.e. ingroup members, neighbors, family, and friends), rather than generalized social trust which is the focus of this study (Freitag and Bauer, 2013). However, due to the non-availability of corresponding indicators, we are not able to study dimensions of particular trust in this study.

^4^The reason for this decision is that moving to another neighborhood or municipality changes individual exposure to segregation patterns, which might be insufficiently captured by our empirical models, especially since the segregation measures we use are municipality-level characteristics. Nonetheless, including movers in the empirical analysis leads to substantively similar conclusions as reported below.

^5^The intra-class correlations (ICC) for the corresponding items in terms of percentages are: Item (i): ICC_individuals_ = 47.2%, ICC_municipalities_ = 4.2%, ICC_municipality−wave_ = 3.4%; Item (ii): ICC_individuals_ = 51.3%, ICC_municipalities_ = 6.4%, ICC_municipality−wave_ = 5.7%.; Item (iii): ICC_individuals_ = 41.7%, ICC_municipalities_ = 2.1%, ICC_municipality−wave_ = 1.8%.; Item (iv): ICC_individuals_ = 42.8%, ICC_municipalities_ = 3.4%, ICC_municipality−wave_ = 2.5%; Item (v): ICC_individuals_ = 40.6%, ICC_municipalities_ = 1.3%, ICC_municipality−wave_ = 0.7%.; Item (vi): ICC_individuals_ = 42.1%, ICC_municipalities_ = 4.4%, ICC_municipality−wave_ = 3.9%.

^6^It is important to note that using a conventional mean index on the three trust items leads to similar results as reported below.

^7^This data is either pre-merged into the NELLS data or was supplemented using the 2009 and 2012 versions of the “Wijk- en Buurtkaart” containing spatial data administered by the Central Bureau of Statistics of the Netherlands. Specifically, we used the following variables to compute proportions and absolute numbers of immigrants (and natives): P_MAROKKO, P_TURKIJE, P_ANT_ARU, P_SURINAM, P_OVER_NW, P_WEST_AL, as well as AANT_INW.

^8^As an example, consider two evenly-populated neighborhoods within a municipality with an overall minority population of 50 out of 100 individuals: if this population lives in a manner so as to be evenly distributed across the two neighborhoods (i.e., 25 in each), the resulting D value would be 0 (i.e., no segregation). However, if 10 minority members live in the first neighborhood, and 40 in the second, this would result in a D value of 0.6, while 50 minority members in one neighborhood, and 0 in the other would lead to a D value of 1 (i.e., full segregation).

^9^In cases in which a municipality consists of only one neighborhood, segregation scores are by definition zero. We excluded these cases from the empirical analysis. It is nonetheless important to note that leaving these municipalities in the analytical sample leads to very similar results, as reported below.

^10^The data on aggregated income also comes from the Wijk- en Buurtkaart. The income thresholds are predefined (variable P_LAAGINKP).

^11^The difference in the correlations across time points (0.22) is not statistically significant: *z* = 1.008, *p* = 0.3137 (Test of equality of two correlation coefficients).

^12^The recoded 7-point response scales of the single items range from 0 (never) to 6 (almost every day). We include this ordinal measure as a continuous predictor in the empirical models for the sake of parsimony. Using dummy variables instead leads to similar results, as reported below. Moreover, the survey also contains items on intergroup friendship. We argue that this measure is prone to a selection issue where trusting individuals systematically select into intergroup friendship, and thus we refrain from including it in our empirical models. It is nonetheless important to note that when all items on intergroup friendship are included in the models, all the main conclusions drawn remain intact.
