# Supplementary material for: Residential Segregation and Social Trust of Immigrants and Natives: Evidence From the Netherlands
Source: Front Sociol. 2020 Jul 7;5:45. doi: 10.3389/fsoc.2020.00045 (PMC8022758; doi:10.3389/fsoc.2020.00045)
Supplement: Supplementary file 1 [file Presentation_1.pdf]

Online Appendix to

Residential Segregation and Social Trust of Immigrants and Natives: Evidence from the Netherlands

Published in *Frontiers in Sociology*:
https://www.frontiersin.org/articles/10.3389/fsoc.2020.00045/abstract

Conrad Ziller, *University of Cologne*

Christoph Spörlein, *Heinrich Heine University Düsseldorf*

Measurement Invariance of Social Trust

An important prerequisite in comparative research is that indicators are comparable across different populations, such as country contexts, sub-groups of respondents, or repeated measurements over time (Davidov et al. 2014). For continuous indicator variables, measurement equivalence is usually tested using multigroup confirmatory factor analysis (MGCFA). Equivalence or measurement invariance typically comprises configural, metric, scalar, and strict invariance. The least demanding version is configural invariance, which requires an equal factor structure across groups. Metric invariance is a little more demanding, requiring equal factor loadings across groups. This means that the same latent construct is measured, and that "an increase of one unit on the measurement scale has the same meaning in population A as in population B" (Davidov et al. 2014, 63). Metric invariance is a requirement for conducting correlational studies and regression analyses with latent factor scores (Hox et al. 2012). Scalar invariance refers to equal indicator loadings and intercepts across groups, which is also required for comparing latent means across groups. Moreover, strict invariance represents identical measurement where also the error structure is equal across groups. In this case, a sum index (instead of a latent variable) can be used.

Model fit is assessed using (changes in) the comparative fit index (CFI), the root mean square error of approximation (RMSEA), and the standardized root mean square residual (SRMR). Model fit can also be assessed using chi-square (difference) tests. However, this measure is

sensitive to sample size, and large samples might lead to the rejection of a model due to trivial discrepancies (Chen 2007, 465).

In this study, measurement equivalence is tested across ethnic groups (natives, immigrants) and across waves (wave1, wave2). Table A1 presents the model fits from a stepwise procedure testing measurement invariance of social trust. Since configural invariance is not testable in the present case with a measurement model of three indicators, we begin with metric invariance for which factor loadings are restricted to equality. To test for scalar invariance, loadings and intercepts are restricted to equality. For tests on strict invariance, errors are additionally restricted to equality. Model fit is assessed is two ways. Generally, a good *absolute* model fit yields a RMSEA smaller than 0.08 (better < 0.05), a CFI greater than 0.90 (better > 0.95), and an SRMR smaller than 0.05 (better < 0.03). *Relative* model fit (comparing subsequent stages of invariance) is assessed using the following cut-off values suggested by Chen (2007, 501): a change in the CFI of less than or equal to -0.01, supplemented by a change in the RMSEA of less than or equal to 0.015, or a change in the SRMR of less than or equal to 0.01 indicates invariance.

Looking at fit statistics for invariance across ethnic groups, metric invariance shows a reasonable model fit. Moving to scalar invariance, model fit worsens slightly, but the RMSEA value sits at the threshold of what can be considered as acceptable. Relaxing the equality constraints for the item "If you help others, you will often be cheated on" (i.e., partial scalar invariance; cf. Byrne et al. 1989) leads to a considerable improvement of the model fit. For strict invariance, model fit statistics indicate that this is not a reasonable assumption. Looking at over-time measurement invariance, model fit statistics even support strict invariance, while partial scalar invariance is strongly supported by the model fit statistics. We draw on the previous literature suggesting that partial invariance with at least two constrained items can be considered a sufficient condition (Steenkamp and Baumgartner 1998). We thus conclude that the indictors used have an equivalent meaning (with some restrictions for one indicator) across ethnic groups and over time, and can thus be employed in regression analysis and for comparing latent means.

Table A1: Measurement Invariance Across Groups and Time

Group: Dutch vs. immigrants	Type of invariance	Chi- square	Chi- square difference	DF difference	p-value difference	RMSEA	CFI	SRMR	RMSEA diff	CFI diff	SRMR diff
	Metric	35.344				0.070	0.993	0.032			
	Scalar	88.743	53.399	2	0.000	0.079	0.983	0.037	0.009	-0.010	0.005
	Partial scalar*	36.781	1.437	1	0.230	0.057	0.993	0.032	-0.013	0.000	0.000
	Strict (Residual)	322.842	234.099	3	0.000	0.115	0.937	0.063	0.036	-0.046	0.026
Group: time (wave2 vs. wave1)	Type of invariance	Chi- square				RMSEA	CFI	SRMR	RMSEA diff	CFI diff	SRMR diff
	Metric	4.827				0.020	1.000	0.013			
	Scalar	19.275	14.448	2	0.000	0.033	0.997	0.013	0.013	-0.003	0.000
	Partial scalar*	4.844	0.017	1	0.898	0.013	0.999	0.013	-0.007	-0.001	0.000
	Strict (Residual)	59.416	40.141	3	0.000	0.047	0.991	0.022	0.014	-0.006	0.009

*For the partial scalar invariance intercepts for indicator variables are constrained to be equal except for indicator (vi) “If you help others, you will often be cheated on” which is allowed to vary. The reference model for partial scalar invariance is metric invariance.

Table A2: Descriptives: Immigrant Background, Wave 1

Variable	Obs	Mean	Std.Dev.	Min	Max
Social trust	617	2.86	.81	1	5
Ethnic segregation	26	.14	.07	.03	.34
Prop. immigrants	140	28.76	15.86	4	79
Age	617	32.21	8.55	15	49
Contact with Dutch	617	4.94	1.18	0	6
Discrimination	617	.61	.49	0	1
Income	617	5.98	2.85	1	16
Unemployed	617	.12	.32	0	1
House ownership	617	.46	.5	0	1
Av. income	26	20.72	1.58	18	23.4
Income segregation	26	.08	.04	.03	.19

Table A3: Descriptives: Immigrant Background, Wave 2

Variable	Obs	Mean	Std.Dev.	Min	Max
Social trust	617	2.8	.88	1	5
Ethnic segregation	26	.21	.11	.05	.55
Prop. immigrants	140	29.96	16.32	4	89
Age	617	36.4	8.56	19.37	53.02
Contact with Dutch	617	4.79	1.2	0	6
Discrimination	617	.57	.5	0	1
Income	617	6.3	2.73	1	16
Unemployed	617	.29	.45	0	1
House ownership	617	.45	.5	0	1
Av. income	26	21.7	1.52	19	24
Income segregation	26	.15	.07	.05	.36

Table A4: Descriptives: Dutch Natives, Wave 1

Variable	Obs	Mean	Std.Dev.	Min	Max
Social trust	795	3.52	.72	1	5
Ethnic segregation	26	.14	.07	.03	.34
Prop. immigrants	160	22.32	14.68	2	71
Age	795	33.36	8.83	15	47
Contact with non-native	795	1.94	1.37	0	6
Income	795	7.32	3.27	1	16
Unemployed	795	.04	.2	0	1
House ownership	795	.81	.39	0	1
Av. income	26	20.72	1.58	18	23.4
Income segregation	26	.08	.04	.03	.19

Table A5: Descriptives: Dutch Natives, Wave 2

Variable	Obs	Mean	Std.Dev.	Min	Max
Social trust	795	3.49	.74	1	5
Ethnic segregation	26	.21	.11	.05	.55
Prop. immigrants	160	23.13	14.85	2	72
Age	795	37.51	8.76	19.53	49.93
Contact with non-native	795	2.44	1.55	0	6
Income	795	7.88	3.08	1	16
Unemployed	795	.09	.29	0	1
House ownership	795	.82	.39	0	1
Av. income	26	21.7	1.52	19	24
Income segregation	26	.15	.07	.05	.36

Table A6: Fixed Effects Regression Results Respondents of Foreign Origin

	(A1)<br>All neighborhoods	(A2)<br>All neighborhoods<br>(with mediators)	(A3)<br>All neighborhoods<br>(with mediators &<br>economic status)	(A4)<br>**Low** concentration<br>neighborhoods	(A5)<br>**High** concentration<br>neighborhoods
Ethnic segregation (munic.)	-0.986**	-1.033**	-1.103**	-1.484	-0.911**
	(0.269)	(0.265)	(0.231)	(1.113)	(0.286)
Prop. immigrants (neigh.)	0.011	0.013	0.013	-0.009	0.012
	(0.008)	(0.008)	(0.008)	(0.020)	(0.008)
Age	-0.052*	-0.050*	-0.048	-0.025	-0.069*
	(0.025)	(0.024)	(0.024)	(0.048)	(0.026)
Contact with Dutch		0.039	0.041		
		(0.038)	(0.038)		
Discrimination		-0.089	-0.091		
		(0.061)	(0.061)		
Income	0.027*	0.026*	0.026*	0.019	0.035
	(0.013)	(0.013)	(0.013)	(0.020)	(0.021)
Unemployed	-0.012	-0.017	-0.019	-0.098	0.036
	(0.091)	(0.090)	(0.092)	(0.158)	(0.112)
House ownership	-0.276	-0.291	-0.317	-0.391	-0.087
	(0.289)	(0.287)	(0.292)	(0.502)	(0.219)
Av. income (munic.)			-0.127		
			(0.121)		
Income segregation (munic.)			0.967		
			(0.874)		
Constant	4.100**	3.871**	6.319**	4.124*	4.261**
	(0.738)	(0.726)	(2.349)	(1.577)	(0.790)
Person fixed effects included	Yes	Yes	Yes	Yes	Yes
Time fixed effects included	Yes	Yes	Yes	Yes	Yes
$N_{observations}$	1234	1234	1234	448	786
$N_{Municipalities}$	26	26	26	21	17
T	2	2	2	2	2

Note: Cluster-robust standard errors in parentheses. * $p < 0.05$, ** $p < 0.01$ (two-sided test).

Table A7: Fixed Effects Regression Results Dutch Respondents

	(A6)<br>All neighborhoods	(A7)<br>All neighborhoods (with mediators)	(A8)<br>All neighborhoods (with mediators & economic status)	(A9)<br>**Low** concentration neighborhoods	(A10)<br>**High** concentration neighborhoods
Ethnic segregation (munic.)	-0.041	-0.017	-0.044	-0.008	-0.114
	(0.217)	(0.205)	(0.201)	(0.201)	(0.337)
Prop. immigrants (neigh.)	0.001	0.001	0.001	-0.008	0.011
	(0.007)	(0.007)	(0.007)	(0.022)	(0.010)
Age	-0.005	-0.005	-0.005	-0.011	0.036
	(0.027)	(0.027)	(0.027)	(0.031)	(0.065)
Contact with non-native		-0.026	-0.027		
		(0.014)	(0.014)		
Income	-0.000	-0.001	-0.001	-0.012	0.020
	(0.012)	(0.012)	(0.012)	(0.012)	(0.029)
Unemployed	-0.068	-0.062	-0.059	0.043	-0.441
	(0.125)	(0.125)	(0.125)	(0.117)	(0.275)
House ownership	-0.022	-0.018	-0.019	-0.291	0.973^{**}
	(0.242)	(0.239)	(0.240)	(0.158)	(0.236)
Av. income (munic.)			0.054		
			(0.063)		
Income segregation (munic.)			-0.645^{*}		
			(0.316)		
Constant	3.683^{**}	3.712^{**}	2.692	4.237^{**}	1.245
	(0.915)	(0.897)	(1.651)	(0.914)	(2.381)
Person fixed effects included	Yes	Yes	Yes	Yes	Yes
Time fixed effects included	Yes	Yes	Yes	Yes	Yes
$N_{observations}$	1590	1590	1590	1190	400
$N_{Municipalities}$	26	26	26	21	16
T	2	2	2	2	2

Note: Cluster-robust standard errors in parentheses. * $p < 0.05$, ** $p < 0.01$ (two-sided test).

Table A8: Four-Level Multilevel Regression Results Respondents of Foreign Origin (**First Generation**)

	(A11)<br>All neighborhoods	(A12)<br>All neighborhoods<br>(with mediators)	(A13)<br>All neighborhoods<br>(with mediators &<br>economic status)	(A14)<br>**Low** concentration<br>neighborhoods	(A15)<br>**High** concentration<br>neighborhoods
Ethnic segregation (munic.)	-0.331	-0.268	-0.392	-0.926	0.008
	(0.378)	(0.347)	(0.408)	(1.111)	(0.435)
Prop. immigrants (neigh.)	-0.008^{**}	-0.008^{**}	-0.008^{**}	-0.000	-0.010^{**}
	(0.003)	(0.003)	(0.003)	(0.015)	(0.003)
Age	-0.014^{**}	-0.013^{**}	-0.014^{**}	-0.004	-0.020^{**}
	(0.005)	(0.005)	(0.005)	(0.009)	(0.006)
Contact with Dutch		0.043	0.044^{*}		
		(0.022)	(0.022)		
Discrimination		-0.000	-0.002		
		(0.058)	(0.058)		
Income	0.041^{**}	0.039^{**}	0.039^{**}	0.031	0.045^{**}
	(0.014)	(0.014)	(0.014)	(0.024)	(0.017)
Unemployed	-0.133	-0.116	-0.123	-0.130	-0.161
	(0.070)	(0.068)	(0.070)	(0.119)	(0.086)
House ownership	0.171^{*}	0.172^{*}	0.171^{*}	0.142	0.180^{*}
	(0.075)	(0.075)	(0.076)	(0.137)	(0.089)
Av. income (munic.)			-0.000		
			(0.031)		
Income segregation (munic.)			0.661		
			(0.746)		
Wave 2	0.023		-0.029	0.064	-0.005
	(0.056)		(0.099)	(0.097)	(0.072)
Constant	3.349^{**}	3.120^{**}	3.167^{**}	2.942^{**}	3.548^{**}
	(0.198)	(0.235)	(0.652)	(0.406)	(0.254)
Random effect municipality	0.035	0.026	0.064^{*}	0.150^{*}	0.077^{*}
Random effect neighborhood	0.123^{**}	0.127^{**}	0.133^{**}	0.339^{**}	0.017
Random effect individual	0.462^{**}	0.459^{**}	0.455^{**}	0.486^{**}	0.396^{**}
Residual	0.629^{**}	0.629^{**}	0.628^{**}	0.569^{**}	0.657^{**}
$N_{Municipalities}$	24	24	24	18	17
$N_{Neighborhoods}$	115	115	115	51	64
$N_{Respondents}$	378	378	378	124	254
$N_{observations}$	756	756	756	248	508

Note: Standard errors in parentheses. * $p < 0.05$, ** $p < 0.01$ (two-sided test).

Table A9: Four-Level Multilevel Regression Results Respondents of Foreign Origin (**Second Generation**)

	(A16)<br>All neighborhoods	(A17)<br>All neighborhoods<br>(with mediators)	(A18)<br>All neighborhoods<br>(with mediators &<br>economic status)	(A19)<br>**Low** concentration<br>neighborhoods	(A20)<br>**High** concentration<br>neighborhoods
Ethnic segregation (munic.)	-1.291*	-1.012	-1.202*	-3.397**	-0.611
	(0.604)	(0.527)	(0.604)	(1.220)	(0.636)
Prop. immigrants (neigh.)	-0.004	-0.004	-0.005	-0.029*	0.005
	(0.004)	(0.004)	(0.004)	(0.014)	(0.006)
Age	-0.000	-0.000	-0.002	0.001	0.005
	(0.007)	(0.007)	(0.007)	(0.009)	(0.010)
Contact with Dutch		0.072*	0.076*		
		(0.033)	(0.033)		
Discrimination		-0.192**	-0.196**		
		(0.071)	(0.071)		
Income	0.048**	0.054**	0.054**	0.043	0.048
	(0.017)	(0.017)	(0.017)	(0.022)	(0.025)
Unemployed	-0.131	-0.093	-0.100	-0.103	-0.128
	(0.112)	(0.111)	(0.111)	(0.160)	(0.153)
House ownership	0.040	0.003	0.014	0.144	-0.147
	(0.095)	(0.094)	(0.094)	(0.130)	(0.132)
Av. income (munic.)			0.064		
			(0.053)		
Income segregation (munic.)			0.640		
			(1.059)		
Wave 2	0.055		-0.054	0.188	-0.022
	(0.065)		(0.132)	(0.099)	(0.087)
Constant	2.963**	2.700**	1.443	3.450**	2.439**
	(0.204)	(0.280)	(1.083)	(0.356)	(0.308)
Random effect municipality	0.267**	0.255**	0.243**	0.330**	0.119
Random effect neighborhood	0.000	0.000**	0.000**	0.191	0.000
Random effect individual	0.523**	0.516**	0.514**	0.421**	0.553**
Residual	0.568**	0.562**	0.562**	0.529**	0.587**
$N_{Municipalities}$	23	23	23	19	13
$N_{Neighborhoods}$	93	93	93	44	49
$N_{Respondents}$	239	239	239	100	139
$N_{observations}$	478	478	478	200	278

Note: Standard errors in parentheses. * $p < 0.05$, ** $p < 0.01$ (two-sided test).

Table A10: Four-Level Multilevel Regression Results Respondents of Foreign Origin (**Turkish Respondents**)

	(A21) All neighborhoods	(A22) All neighborhoods (with mediators)	(A23) All neighborhoods (with mediators & economic status)	(A24) **Low** concentration of Turks neighborhoods	(A25) **High** concentration of Turks neighborhoods
Ethnic segregation (munic.)	0.004	0.027	0.129	-0.350	0.598
	(0.454)	(0.413)	(0.456)	(0.655)	(0.588)
Prop. Turks (neigh.)	-0.028**	-0.027**	-0.025**	-0.199*	-0.026**
	(0.007)	(0.007)	(0.007)	(0.080)	(0.008)
Age	-0.011*	-0.010	-0.009	-0.016	-0.008
	(0.005)	(0.005)	(0.006)	(0.010)	(0.007)
Contact with Dutch		0.076*	0.077**		
		(0.030)	(0.030)		
Discrimination		-0.047	-0.043		
		(0.074)	(0.074)		
Income	0.042**	0.043**	0.042**	0.043	0.036
	(0.016)	(0.016)	(0.016)	(0.024)	(0.021)
Unemployed	-0.053	-0.031	-0.027	-0.050	-0.062
	(0.104)	(0.103)	(0.104)	(0.168)	(0.131)
House ownership	0.089	0.069	0.072	0.209	0.033
	(0.092)	(0.092)	(0.092)	(0.162)	(0.109)
Av. income (munic.)			0.010		
			(0.029)		
Income segregation (munic.)			-0.773		
			(0.881)		
Wave 2	-0.003		0.058	0.055	-0.049
	(0.068)		(0.114)	(0.107)	(0.088)
Constant	2.995**	2.598**	2.382**	3.412**	2.874**
	(0.187)	(0.255)	(0.657)	(0.362)	(0.221)
Random effect municipality	0.000**	0.000**	0.000	0.000**	0.000
Random effect neighborhood	0.125*	0.098	0.065	0.000**	0.000**
Random effect individual	0.455**	0.464**	0.467**	0.524**	0.419**
Residual	0.660**	0.652**	0.653**	0.586**	0.692**
$N_{Municipalities}$	20	20	20	18	17
$N_{Neighborhoods}$	81	81	81	37	64
$N_{Respondents}$	255	255	255	84	171
$N_{observations}$	510	510	510	168	342

Note: Standard errors in parentheses. * $p < 0.05$, ** $p < 0.01$ (two-sided test).

Table A11: Four-Level Multilevel Regression Results Respondents of Foreign Origin (**Moroccan Respondents**)

	(A26) All neighborhoods	(A27) All neighborhoods (with mediators)	(A28) All neighborhoods (with mediators & economic status)	(A29) **Low** concentration of Moroccans neighborhoods	(A30) **High** concentration of Moroccans neighborhoods
Ethnic segregation (munic.)	-1.466**	-1.376**	-1.667**	-2.531**	-0.931
	(0.395)	(0.365)	(0.422)	(0.686)	(0.489)
Prop. Moroccans (neigh.)	-0.012	-0.012	-0.012	-0.266**	-0.011
	(0.006)	(0.006)	(0.007)	(0.087)	(0.007)
Age	-0.008	-0.007	-0.007	-0.031**	0.002
	(0.005)	(0.005)	(0.005)	(0.009)	(0.006)
Contact with Dutch		0.002	0.004		
		(0.027)	(0.027)		
Discrimination		0.044	0.043		
		(0.069)	(0.069)		
Income	0.061**	0.061**	0.059**	0.076**	0.045*
	(0.018)	(0.018)	(0.018)	(0.028)	(0.022)
Unemployed	-0.219**	-0.212**	-0.219**	-0.058	-0.287**
	(0.082)	(0.080)	(0.082)	(0.136)	(0.097)
House ownership	0.227*	0.226*	0.226*	0.287	0.242*
	(0.099)	(0.099)	(0.100)	(0.168)	(0.119)
Av. income (munic.)			-0.015		
			(0.043)		
Income segregation (munic.)			1.514		
			(0.844)		
Wave 2	0.033		-0.074	0.132	-0.004
	(0.062)		(0.110)	(0.092)	(0.079)
Constant	3.080**	3.029**	3.339**	4.241**	2.759**
	(0.183)	(0.246)	(0.888)	(0.351)	(0.235)
Random effect municipality	0.000	0.000	0.000	0.163	0.000
Random effect neighborhood	0.144*	0.135*	0.113	0.000**	0.000
Random effect individual	0.443**	0.446**	0.450**	0.551**	0.360**
Residual	0.581**	0.581**	0.578**	0.470**	0.623**
$N_{Municipalities}$	21	21	21	19	10
$N_{Neighborhoods}$	83	83	83	41	42
$N_{Respondents}$	249	249	249	82	167
$N_{observations}$	498	498	498	164	334

Note: Standard errors in parentheses. * $p < 0.05$, ** $p < 0.01$ (two-sided test).

References

Chen, F. F. (2007). Sensitivity of goodness of fit indexes to lack of measurement invariance. *Structural equation modeling*, 14(3), 464–504.

Davidov, E., Meuleman, B., Cieciuch, J., Schmidt, P., Billiet, J. (2014). Measurement equivalence in cross-national research. *Annual Review of Sociology*, *40*, 55–75.

Hox J.J., de Leeuw E.D., Brinkhuis M.J., Ooms J. (2012). Multigroup and multilevel approaches to measurement equivalence. In *Methods, Theories, and Empirical Applications in the Social Sciences,* ed. S Salzborn, E Davidov, J Reinecke, pp. 91–96. Wiesbaden: Springer VS.

Byrne, B. M., Shavelson, R. J., Muthén, B. (1989). Testing for the equivalence of factor covariance and mean structures: the issue of partial measurement invariance. *Psychol. Bull.* 105, 456–466.

Steenkamp, J.-B. E. M., Hans Baumgartner, H. (1998). Assessing measurement invariance in cross-national consumer research. *Journal of Consumer Research*, 25(1), 78–90.
